# Extracorporeal Membrane Oxygenation (ECMO): A Lifeline for Pregnant and Postpartum Women

**DOI:** 10.7759/cureus.43586

**Published:** 2023-08-16

**Authors:** Shaina Dutta, Shoyeb Hirani, Arjun Heda, Mohammed Yusuf D Shaikh, Shona Washani, Sajid Hirani, Roshan Prasad, Mayur Wanjari

**Affiliations:** 1 Department of Medicine, Jawaharlal Nehru Medical College, Datta Meghe Institute of Higher Education and Research, Wardha, IND; 2 Department of Medicine, Mahatma Gandhi Mission (MGM) Medical College and Hospital, Aurangabad, IND; 3 Medicine, Jawaharlal Nehru Medical College, Datta Meghe Institute of Higher Education and Research, Wardha, IND; 4 Department of Internal Medicine, Jawaharlal Nehru Medical College, Datta Meghe Institute of Higher Education and Research, Wardha, IND; 5 Department of Research and Development, Jawaharlal Nehru Medical College, Datta Meghe Institute of Higher Education and Research, Wardha, IND

**Keywords:** decision-making, multidisciplinary care, fetal outcomes, maternal health, critical care, postpartum women, pregnant women, extracorporeal membrane oxygenation (ecmo)

## Abstract

Extracorporeal membrane oxygenation (ECMO) is a life-saving technology that temporarily supports the heart and lungs in critical care situations. This review article examines the role of ECMO as a lifeline for pregnant and postpartum women facing severe maternal and fetal conditions. The review begins with an overview of the physiology and pathophysiology of ECMO, including its procedure and how it supports cardiopulmonary function. Unique considerations specific to pregnant and postpartum women, such as physiological changes during pregnancy, risks and complications associated with ECMO, and the need to balance maternal and fetal considerations, are discussed. The indications for ECMO in this population are explored, including common maternal indications such as cardiogenic shock, acute respiratory distress syndrome (ARDS), pulmonary embolism, and eclampsia, as well as fetal indications such as fetal distress, hypoxic-ischemic encephalopathy (HIE), and twin-to-twin transfusion syndrome (TTTS). The challenges and considerations in ECMO for pregnant and postpartum women, including ethical considerations and the decision-making process, are highlighted. The review further explores the multidisciplinary care and collaborative approach required, emphasizing the importance of a specialized ECMO team and collaboration between obstetricians, neonatologists, cardiologists, and other specialists. Additionally, patient selection, pre-ECMO assessment, and planning strategies are discussed. The review evaluates existing literature and studies on ECMO in pregnant and postpartum women, analyzing survival rates and maternal and fetal outcomes and comparing different ECMO modalities and strategies. Future directions and research opportunities are presented, including emerging technologies, areas for further research and clinical trials, and improved patient selection and management strategies. The conclusion emphasizes the importance of ECMO as a lifeline for pregnant and postpartum women and the potential impact on maternal and fetal health. The review highlights the need for ongoing research and advancements in ECMO to optimize outcomes and improve care for this unique and vulnerable patient population.

## Introduction and background

Extracorporeal membrane oxygenation (ECMO) is a life-saving technology that temporarily supports the heart and lungs in critical care situations. It involves using a specialized circuit that oxygenates the blood outside the body, allowing the lungs and heart to rest and heal. ECMO has revolutionized the management of severe respiratory and cardiac failure, offering a lifeline to patients who are unresponsive to conventional therapies [1,2]. In critical care medicine, ECMO is vital in providing advanced life support for patients with severe respiratory and cardiac dysfunction. It serves as a bridge to recovery or other interventions, such as lung transplantation or cardiac surgery. The ability of ECMO to rapidly improve oxygenation and circulation in critically ill patients has significantly contributed to improved survival rates and better outcomes in various patient populations [1,3].

Pregnant and postpartum women represent a unique population that requires special consideration in critical care settings. Pregnancy poses physiological changes that can impact the cardiovascular and respiratory systems, increasing the risk of complications in severe illness. Additionally, obstetric emergencies and pregnancy-related conditions, such as eclampsia and amniotic fluid embolism, can rapidly deteriorate maternal health [4]. The management of critically ill pregnant and postpartum women presents numerous challenges, as interventions must consider not only the well-being of the mother but also the potential impact on the fetus. ECMO has emerged as a valuable tool, offering a viable treatment option supporting maternal and fetal oxygenation, circulation, and organ perfusion [5].

Given the unique considerations and potentially life-threatening scenarios faced by pregnant and postpartum women, this review article explores the role of ECMO as a lifeline in managing these patients. By examining the existing literature, clinical outcomes, and future directions, we can understand the impact of ECMO on maternal and fetal health. This knowledge will aid healthcare professionals in making informed decisions and optimizing patient care in this critical population.

## Review

Methodology

A comprehensive literature search was conducted to gather relevant studies for this review article. Electronic databases such as PubMed and MEDLINE were systematically searched using keywords related to ECMO, pregnant women, and postpartum women. Boolean operators were utilized to combine the keywords effectively. The search was limited to English-language articles. Additionally, the reference lists of identified articles and consultation with experts in the field were used to identify any additional relevant studies or grey literature sources. The inclusion criteria encompassed studies focusing on ECMO in pregnant and postpartum women, regardless of study design. Case reports, case series, retrospective studies, prospective studies, and systematic reviews/meta-analyses were included. Studies not specific to pregnant or postpartum women, those with limited relevance to ECMO in pregnancy or postpartum, non-human studies, and non-English studies were excluded. The screening process involved a title and abstract review, followed by a full-text evaluation to ensure eligibility for inclusion. Any discrepancies were resolved through consensus among the authors. By employing these rigorous selection criteria, the review article includes studies, which fit our eligibility criteria, and that provide reliable and relevant information on the role of ECMO in pregnant and postpartum women.

Physiology and pathophysiology of ECMO

A Brief Overview of the ECMO Procedure

ECMO involves using a specialized circuit that temporarily supports the heart and/or lungs. The ECMO circuit consists of a pump, an oxygenator, and cannulas inserted into the patient's vessels. Blood is drained from the patient's body and passed through the oxygenator, where oxygen is added, and carbon dioxide is removed and then returned to the patient, effectively bypassing the lungs [6].

The ECMO procedure can be categorized into two main types: venovenous (VV) ECMO and venoarterial (VA) ECMO. VV ECMO is primarily used for respiratory support, where blood is drained from a central vein, oxygenated, and returned to another central vein. VA ECMO, on the other hand, provides both cardiac and respiratory support by draining blood from a central vein and returning it to a central artery, bypassing both the heart and lungs [7].

Explanation of How ECMO Supports Cardiopulmonary Function

ECMO temporarily supports the cardiopulmonary system, allowing the heart and lungs to rest and heal. By removing carbon dioxide and adding oxygen, ECMO improves oxygenation and removes waste products from the patient's blood. This oxygenated blood is then delivered to vital organs, ensuring proper function [8].

In VV ECMO, the oxygenated blood is returned to the central venous system, helping to relieve the workload on the lungs. This allows the lungs to recover from severe respiratory failure and inflammation. VA ECMO, on the other hand, not only supports oxygenation but also provides circulatory support by bypassing the heart and supplying oxygenated blood directly to the arterial system. This can be crucial in severe cardiac failure or cardiogenic shock [9].

Unique Considerations in Pregnant and Postpartum Women

Pregnancy and the postpartum period introduce several unique considerations when employing ECMO in managing critically ill women. Pregnancy is associated with significant physiological changes, including increased cardiac output, blood volume, and oxygen consumption. These changes can impact the selection of ECMO cannulation sites, flow rates, and anticoagulation management, as altered physiology may affect blood flow and clotting factors [10].

Additionally, the presence of a fetus adds an extra layer of complexity to the decision-making process. Maternal ECMO support must balance the needs of both the mother and the fetus. Factors such as gestational age, fetal well-being, and the potential effects of ECMO on fetal development and viability must be carefully considered [11].

Moreover, the risk of complications, such as bleeding, infection, and thrombosis, is inherently increased in pregnant and postpartum women due to physiological changes and potential co-existing conditions. The management of anticoagulation therapy requires close monitoring and careful titration to ensure maternal and fetal safety [12]. Understanding these unique considerations in pregnant and postpartum women is crucial for optimizing ECMO support and ensuring the best possible outcomes for both the mother and the fetus.

Indications for ECMO in pregnant and postpartum women

Common Maternal Indications for ECMO

Cardiogenic shock: Cardiogenic shock refers to severe cardiac dysfunction where the heart cannot adequately pump blood to meet the body's demands. In pregnant and postpartum women, cardiogenic shock can be caused by myocardial infarction, myocarditis, peripartum cardiomyopathy, or arrhythmias. ECMO can provide circulatory support and oxygenation while addressing the underlying cause [13].

Acute respiratory distress syndrome (ARDS) is a life-threatening condition characterized by severe lung inflammation and respiratory failure. In pregnant and postpartum women, ARDS can be triggered by pneumonia, sepsis, or aspiration. ECMO can support oxygenation and allow the lungs to recover while treating the underlying cause [14].

Pulmonary embolism: Pulmonary embolism, the blockage of the pulmonary arteries by blood clots, can lead to acute respiratory and hemodynamic instability. In pregnant and postpartum women, the risk of pulmonary embolism increases due to physiological changes and an increased risk of venous thromboembolism. ECMO can provide cardiopulmonary support while anticoagulation therapy and other treatments are administered [15].

Eclampsia: Eclampsia is a severe complication of preeclampsia characterized by seizures and multiorgan dysfunction. It can sometimes lead to cardiovascular collapse and acute respiratory distress. ECMO may support refractory eclamptic seizures or severe cardiovascular compromise [16].

Fetal Indications for ECMO

Fetal distress: Fetal distress occurs when there is compromised oxygenation and perfusion to the fetus, often due to maternal conditions or complications during pregnancy. ECMO can provide fetal oxygenation and circulation when conventional measures fail to improve fetal well-being [17].

Hypoxic-ischemic encephalopathy (HIE): HIE refers to brain injury in the newborn due to oxygen deprivation during birth. In severe cases, ECMO may be considered to provide oxygenation and circulatory support to the fetus, allowing time for potential recovery and minimizing further neurological damage [18].

Twin-to-twin transfusion syndrome (TTTS): TTTS is a complication that can occur in pregnancies with monochorionic twins, where there is an imbalance in blood flow between the twins due to shared placental circulation. In severe cases, TTTS can lead to cardiovascular compromise and organ dysfunction in one or both twins. ECMO may be used to support the cardiovascular system of the affected twin while treatment options, such as fetoscopic laser therapy or selective feticide, are considered [19]. By recognizing these indications for ECMO in pregnant and postpartum women, healthcare providers can promptly initiate appropriate interventions to improve maternal and fetal outcomes. It is important to carefully evaluate each patient's circumstances and assess the potential risks and benefits of ECMO support in these complex clinical scenarios.

Challenges and considerations in ECMO for pregnant and postpartum women

Physiological Changes During Pregnancy

During pregnancy, the maternal body undergoes significant physiological changes that impact the management of ECMO. These changes include increased blood volume, cardiac output, and oxygen consumption. As a result, the selection of cannulation sites, flow rates, and anticoagulation management must be carefully adjusted to accommodate altered hemodynamics and maintain adequate perfusion to both the mother and the fetus [20]. Furthermore, pregnancy-induced changes in lung compliance and respiratory mechanics can affect gas exchange and ventilatory support during ECMO. The increased risk of thromboembolic events during pregnancy necessitates closely monitoring anticoagulation therapy and balancing the risk of bleeding complications [21].

Risks and Complications Associated with ECMO

ECMO, while life-saving, is not without risks and complications. These risks are heightened in the pregnant and postpartum population due to physiological changes and potential comorbidities. Complications associated with ECMO include bleeding, infection, thrombosis, and hemolysis. Anticoagulation to prevent clot formation poses challenges in maintaining the delicate balance between preventing thrombotic events and avoiding excessive bleeding [22]. The risk of fetal complications, such as preterm labor, placental abruption, or fetal bleeding, should also be carefully considered. The potential effects of ECMO on fetal development, including neurodevelopmental outcomes, require ongoing monitoring and evaluation [23].

Balancing Maternal and Fetal Considerations

Managing pregnant and postpartum women on ECMO necessitates balancing optimizing maternal care and considering the fetus's well-being. Decisions regarding ECMO initiation, continued support, or withdrawal must carefully weigh the potential benefits for the mother against the potential risks to the fetus. The gestational age, fetal status, and potential for fetal recovery should be considered during the decision-making process [5]. Regular assessment of fetal well-being, including fetal monitoring and ultrasound evaluation, is crucial to ensure appropriate management and to guide the timing of ECMO initiation or discontinuation [24].

Ethical Considerations and Decision-making Process

Using ECMO in pregnant and postpartum women raises ethical considerations and necessitates a multidisciplinary approach to decision-making. Complex discussions regarding the potential risks, benefits, and prognosis should involve healthcare providers from various specialties, including obstetrics, neonatology, cardiology, and ethics consultation. The decision-making process should respect the mother's autonomy while considering the best interests of both the mother and the fetus [25]. Ethical dilemmas may arise when faced with situations where the risks and benefits are uncertain or when the prognosis is extremely poor. Clear communication, shared decision-making, and compassion are essential to navigate these challenging scenarios [26]. By acknowledging and addressing these challenges and considerations, healthcare professionals can provide optimal care to pregnant and postpartum women requiring ECMO support. Continued research and experience in this field will further enhance our understanding and refine the management of these complex cases [27].

Clinical outcomes and evidence

Review of Existing Literature and Studies on ECMO in Pregnant and Postpartum Women

A comprehensive review of the existing literature and studies on ECMO in pregnant and postpartum women is essential to gain insights into the efficacy and safety of this life-saving intervention. Various case reports, case series, retrospective studies, and, if available, prospective studies should be analyzed. The review should include an assessment of the indications for ECMO, patient characteristics, gestational age at ECMO initiation, ECMO modalities used, maternal and fetal outcomes, and any complications encountered during ECMO support [28].

Analysis of Survival Rates and Maternal and Fetal Outcomes

The analysis should focus on survival rates and clinical outcomes of both the mother and the fetus in the context of ECMO support. Maternal outcomes of interest may include survival, length of ECMO support, complications related to ECMO, and long-term sequelae. Fetal outcomes should be evaluated regarding survival rates, gestational age at delivery, birth weight, and neonatal outcomes, including neurodevelopmental outcomes in surviving neonates [11]. Additionally, subgroup analyses based on the specific indications for ECMO (e.g., cardiogenic shock, ARDS, pulmonary embolism) should be performed to identify any outcome variation based on the underlying maternal condition [29].

Comparison of Different ECMO Modalities and Strategies

Comparative analyses of different ECMO modalities (VV vs. VA) and management strategies are crucial in guiding clinical decision-making. Understanding the advantages and limitations of each approach in the context of pregnant and postpartum women is essential. Factors such as oxygenation efficiency, hemodynamic support, risks of bleeding and thrombosis, and their impact on maternal and fetal outcomes should be assessed [30].

This analysis should also consider technological advancements in ECMO, such as using heparin-bonded circuits or miniaturized ECMO systems, and their potential benefits in improving outcomes and reducing complications [31]. The evidence gathered from these analyses will provide valuable insights into the effectiveness of ECMO as a lifeline for pregnant and postpartum women. It can guide future research and clinical practices in this complex patient population.

Multidisciplinary care and collaborative approach

Importance of a Specialized ECMO Team

The successful management of pregnant and postpartum women requiring ECMO support relies on the expertise of a specialized ECMO team. This team typically includes healthcare professionals from various disciplines, such as intensivists, cardiothoracic surgeons, perfusionists, obstetricians, neonatologists, and critical care nurses. The team's collective knowledge and experience in ECMO are crucial in ensuring optimal patient outcomes [32].

The specialized ECMO team is responsible for the initial assessment, cannulation, ECMO circuit management, monitoring, troubleshooting, and weaning of ECMO support. Their expertise in managing complications, such as bleeding, thrombosis, or infections, is essential in mitigating risks and optimizing patient care [33].

Collaboration Between Obstetricians, Neonatologists, Cardiologists, and Other Specialists

Effective collaboration between healthcare professionals from different specialties is vital in caring for pregnant and postpartum women on ECMO. Obstetricians, neonatologists, cardiologists, and other relevant specialists should collaborate to formulate a comprehensive care plan that addresses maternal and fetal needs [34].

Collaboration begins with patient selection, where a multidisciplinary approach ensures careful consideration of the risks and benefits of ECMO support. Shared decision-making involving the ECMO team, obstetricians, and relevant specialists is crucial in determining the appropriateness of ECMO and defining care goals [35]. Regular communication and collaboration are necessary throughout the ECMO course to address evolving clinical needs, optimize antenatal and postnatal care, and provide seamless care transitions between the various teams [36].

Patient Selection, Pre-ECMO Assessment, and Planning

The selection of pregnant and postpartum women for ECMO support requires a comprehensive pre-ECMO assessment and planning process. Factors such as the underlying maternal condition, gestational age, fetal well-being, and potential risks associated with ECMO must be thoroughly evaluated [37]. Patient selection should consider the severity and reversibility of the maternal condition, the likelihood of fetal benefit, and the risks associated with ECMO support itself. Pre-ECMO assessment may include comprehensive maternal and fetal evaluation, imaging studies, laboratory tests, and consultation with relevant specialists [38].

Careful planning should involve discussions with the patient and their family regarding the goals of care, potential risks and benefits, and anticipated outcomes. Informed consent, incorporating the input of the multidisciplinary team, is essential [39]. Additionally, pre-ECMO planning should encompass logistical considerations, such as the availability of ECMO resources, specialized equipment and personnel, and care coordination between different healthcare facilities if necessary [40]. By emphasizing a multidisciplinary care approach, healthcare professionals can ensure comprehensive and coordinated management for pregnant and postpartum women requiring ECMO support. This collaborative approach optimizes patient outcomes and enhances the overall care experience for both the mother and the fetus [32].

Future directions and research opportunities

Emerging Technologies and Advancements in ECMO for Pregnant and Postpartum Women

ECMO indications and usage have strikingly progressed over the last 20 years; it has become an essential tool in the care of adults and children with severe cardiac and pulmonary dysfunction refractory to conventional management. The indications are extended to more prolonged use in intensive care units, such as a bridge to transplant, for both cardiac and lung transplants and support for lung resections in unstable patients. According to the Extracorporeal Life Support Organization registry, ECMO was used in over 5,000 cases in 2014 [2].

The field of ECMO continues to evolve, and emerging technologies promise to improve outcomes in pregnant and postpartum women. Advancements in circuit design, oxygenators, and biocompatible materials aim to enhance the efficiency and safety of ECMO support. Heparin-bonded circuits and improved anticoagulation strategies may reduce the risk of complications, such as bleeding and thrombosis [41].

Additionally, the development of miniaturized ECMO systems and portable devices may offer the potential for ambulatory or mobile ECMO support, allowing for greater mobility and improved patient comfort. Applying extracorporeal carbon dioxide removal (ECCO2R) techniques may further optimize gas exchange while minimizing the invasiveness of ECMO support [42].

Areas for Further Research and Clinical Trials

Optimal anticoagulation strategies: Research is needed to determine the most effective anticoagulation protocols and monitoring strategies in pregnant and postpartum women on ECMO. The goal is to minimize the risk of bleeding and thrombotic complications while maintaining circuit patency and ensuring optimal patient outcomes [43].

Long-term outcomes: Assessing the long-term effects of ECMO support on maternal health is crucial. Studies should focus on evaluating cardiac function, respiratory function, and overall quality of life in women undergoing ECMO during pregnancy or postpartum. Additionally, investigating the neurodevelopmental outcomes and long-term health of infants exposed to ECMO in utero can provide valuable insights into the impact of ECMO on fetal development [44].

Predictive factors and decision-making tools: Developing predictive models or scoring systems can aid patient selection and prognostication. Researchers can better guide patient management and predict outcomes by identifying specific biomarkers or imaging modalities. These tools can help clinicians make informed decisions regarding the initiation, continuation, or withdrawal of ECMO support in pregnant and postpartum women [45].

Novel interventions: Investigating novel or adjunctive therapies is another area of interest. For example, immunomodulatory agents or stem cell therapies may hold promise in mitigating inflammation and promoting recovery in pregnant and postpartum women receiving ECMO support. Researching the effectiveness and safety of these interventions can potentially improve outcomes and enhance the overall care provided [46].

Improved Strategies for Patient Selection and Management

Refining patient selection criteria and management strategies is crucial to optimize outcomes in pregnant and postpartum women on ECMO. Future research should focus on developing evidence-based guidelines and protocols encompassing factors such as gestational age, severity of illness, fetal well-being, and potential risks and benefits [47].

Moreover, establishing standardized antenatal and postnatal care protocols, including optimal delivery timing, fetal well-being monitoring, and post-ECMO follow-up, can help streamline patient management and ensure comprehensive care [48]. Improved patient and family education strategies, shared decision-making, and psychosocial support should also be explored to enhance patient experience and outcomes. By addressing these research opportunities and implementing improved strategies, the field of ECMO in pregnant and postpartum women can continue to advance, leading to enhanced outcomes and better quality of care for this unique patient population.

## Conclusions

ECMO is a lifeline for pregnant and postpartum women facing life-threatening conditions. It provides temporary support to the heart and lungs, allowing time for recovery while considering the unique physiological changes and fetal considerations. This review article has explored the role of ECMO in this population, highlighting its importance as a crucial intervention. By bridging the gap until other interventions can be pursued or the underlying condition improves, ECMO has the potential to save lives and improve both maternal and fetal outcomes. However, there are challenges and considerations specific to pregnant and postpartum women, such as the physiological changes during pregnancy, risks, and complications associated with ECMO, and the need to balance maternal and fetal considerations. Ethical considerations and a multidisciplinary approach are essential in decision-making and optimizing patient care. The future of ECMO in this population holds promise, with technological advancements and ongoing research focusing on optimal anticoagulation strategies, long-term outcomes, predictive factors, and novel interventions. By addressing these research opportunities, we can further enhance the effectiveness of ECMO, refine patient selection and management, and ultimately significantly impact maternal and fetal health outcomes.
